# Clinical Application of Atlantoaxial Pedicle Screw Placement Assisted by a Modified 3D-Printed Navigation Template

**DOI:** 10.6061/clinics/2018/e259

**Published:** 2018-07-13

**Authors:** Xingwei Pu, Chunshan Luo, Tinsheng Lu, Shudan Yao, Qiling Chen

**Affiliations:** Department of Spinal Surgery, Guizhou Orthopedic Hospital, Guiyang, 550002, China

**Keywords:** Atlantoaxial, Improvement, Navigation Template, 3D Print, Pedicle Screw

## Abstract

**OBJECTIVES:**

To investigate the primary clinical value of atlantoaxial pedicle screw placement assisted by a modified 3D-printed navigation template.

**METHODS:**

We retrospectively analyzed the cases of 17 patients treated from June 2015 to September 2016 with atlantoaxial pedicle screw placement assisted by a modified 3D-printed navigation template. All procedures were performed prior to surgery, including thin-slice CT scanning, medical image sampling and computerized 3D modeling of the atlantoaxial joint, optimal pedicle screw trajectory determination, and anatomical trait acquisition for the atlantoaxial pedicle, spinous process of the axis, vertebral lamina and posterior lateral mass, and design of a reverse template. During surgery, a navigation template was tightly attached to the atlantoaxial joint to assist in pedicle screw placement. Surgeons subsequently used an electric drill to remove the template through a guide channel and then placed the atlantoaxial pedicle screw. Observed indexes included the VAS score, JOA improvement rate, surgery duration, and blood loss.

**RESULTS:**

Surgery was successful in all 17 patients, with an average operation duration of 106±25 min and an average blood loss of 220±125 ml. Three days postoperatively, the VAS score decreased from 6.42±2.21 to 3.15±1.26. Six months postoperatively, the score decreased to 2.05±1.56. The postoperative JOA score increased significantly from 7.68±2.51 to 11.65±2.72 3 d after surgery and to 13.65±2.57 after 6 months. Sixty-eight pedicle screws were inserted successfully, with 34 in the atlas and 34 in the axis. According to the Kawaguchi standard, 66 screws were in grade 0 (97.06%), and 2 were in grade 1 (2.94%). The pre- and postoperative transverse and sagittal screw angles showed no significant differences.

**CONCLUSIONS:**

Atlantoaxial pedicle screw placement assisted by a modified 3D-printed navigation template is worth recommending due to the improved accuracy in screw placement, improved patient safety and beneficial clinical effects.

## INTRODUCTION

Posterior pedicle screw fixation of the atlantoaxial joint is a widely recognized treatment option by spinal surgeons due to its advantages of long-term stability, high fusion rates, and good position correction 1. This technique is the gold standard treatment for atlantoaxial dislocation and instability due to surgical treatment. However, the procedure’s application is greatly limited due to the high risk of damage to vascular and nerve tissue, the small size of the pedicle, the high mutational rate and the complex structure of the atlantoaxial surroundings 2. Thus, screw placement has become an important factor of successful surgery, and improving the accuracy of atlantoaxial pedicle screw placement has become an urgent need. In recent years, a 3D printing technique using a pedicle navigation template has been used in spinal surgery 3-5. Nevertheless, small templates may be difficult to use in fixation and may result in screw placement deviation due to template shifting 6. These templates are fragile and inclined to be unstable in application; additionally, disinfection and sterilization are likely to cause template deformation. Therefore, we modified the small template, designed a new template using reverse engineering software and a 3D printing technique, and applied the modified navigation template in atlantoaxial dislocation and deformity treatments from June 2015 to September 2016. The clinical effect was beneficial and is summarized herein.

## MATERIALS AND METHODS

### Materials

From June 2015 to September 2016, 17 patients with atlantoaxial dislocation or deformity were treated in our department and subsequently followed for 6-8 months. There were 11 males and 6 females ranging in age from 25 to 56 years with a mean age of 43.3 years. Of the 17 patients, 13 were treated for atlantoaxial dislocation, and 4 were treated for atlantoaxial deformity. All patients suffered from neck and shoulder pain, including 5 patients with accompanying cervical cord compression. Imaging examination showed that all 17 patients suffered from cervical instability or dislocation. Fifteen of the patients could fully recover before the surgery, while the other 2 could only generally recover. After surgery, there was no obvious pressure on the anterior aspect of the spinal cord. This study was approved by the ethics committee.

### Design and production of a modified 3D-printed navigation template

We used a LightSpeed 16-slice spiral CT machine (GE, USA) for the imaging examinations. The scanning range included three segments from the first cervical vertebra to the third cervical vertebra with a 1.25-mm slice thickness, 5.0-mm pitch, 9.37-mm/s table speed, 120-kV voltage, and 200-mA current. Scanned images were saved in DICOM format, and the DICOM files were imported into the 3D image producing and editing software Mimics 17.0 (Materialise, Belgium) to construct 3D models of the target cervical area. To avoid damage in the trajectory to the pedicle cortex, preliminary simulation of pedicle screw placement was conducted with computer-aided design software Creo2.0 (PTC, America) using a cylindrical simulated screw with a diameter of 3.5 mm to observe the positional relationship between the trajectory and pedicle in the transverse, sagittal and coronal views of the 3D image. The axis of the cylinder 3.5 mm in diameter was used to build a screw placement guide tube with an inner diameter of 2.1 mm and a vertebral lamina with a length of 15 mm. The template location surface was formed by removing the interfering parts of the template and 3D model after designation of the screw hole position. A cylindrical range pole 3.5 mm in diameter was rebuilt based on the preset axis line of the screw hole. The range pole was moved approximately 10 mm toward the screw hole, and the average thickness of the template was set at approximately 6 mm. The template file was imported into a Formlab 3D printer (Formlabs, USA) to print the cervical pedicle screw placement template. Ethylene oxide was subsequently used to disinfect the template, which was later sealed for future use.

### Clinical application of modified 3D-printed template

A preoperative simulation surgery was conducted using the 3D-printed template in this study. Kirschner wire was drilled through the guide channel of the modified 3D-printed navigation template and the pedicle to ensure that the wire was completely in the pedicle and would not break the pedicle cortex. *In vitro* testing demonstrated the accuracy of the modified 3D-printed navigation template. After feasibility of the treatment was assured, the navigation template was disinfected with ethylene oxide before use on patients. All patients maintained an atlantoaxial reduction position with a skull traction weight of 3-6 kg. After receiving general anesthesia, patients in the prone position were incised in the middle of neck to expose the posterior atlantoaxial region with complete dissection of the muscles and ligaments attached to the lamina and spinous process. The disinfected modified 3D-printed navigation template was later attached to the lateral mass, lamina, and spinous process of the corresponding vertebra. Next, after close observation, the surgeons drilled into the vertebral bone cortex to a depth of 10 mm with an electric drill and a 2.0-mm-diameter bit, and assistants subsequently attached the template. Parallel with the direction of the guide posts, the electric drill was used in the guide channels to investigate optimum screw placement and to observe the post’s direction and movement. Probes were used after drilling to detect whether the internal walls of the screw channels were intact. Threads and pedicle screws with a diameter of 3.5 mm were placed once the intact screw channels were confirmed. After screw placement and lateral observation using a C-arm X-ray machine, the screw position was confirmed, and the surgeons kept patients in the optimum reduction position after adjusting their heads and necks. Bone grafting was completed after screw-rod fixation. Finally, drainage tubes were inserted, and the incision was sutured outward.

### Postoperative management

After surgery, patients were kept under strict bed rest with the cervical spine held stationary and were turned over axially as needed. The drainage flow of the operative site was observed, and the drainage tube was removed if the drainage flow was less than 50 ml/24 h without cerebrospinal fluid leakage. Before surgery, the patients had taken an antibiotic for 24-48 h, a dehydrant for 3-5 d, and a gastric acid suppression agent for 3-5 d. Adrenalineand was added for 3-5 d if there was impaired spinal cord function before surgery. Patients wore a cervical support device to perform bedside exercises 3 d after surgery.

### Assessment

Regular atlantoaxial CT scanning was used to observe the position of the pedicle screw. The accuracy of screw placement was assessed with the Kawaguchi standard by observing the positional relationship between the screw and pedicle, as follows: Level 0: the screw is completely in the pedicle; Level 1: the screw is out of the cortex of the pedicle by less than 2 mm, and no screw-related complications occurred, including nerve or vertebral artery injury; Level 2: the screw is out of the cortex of the pedicle by more than 2 mm, and no screw-related complications occurred, including nerve or vertebral artery injury; Level 3: Screw-related complications occurred, including nerve or vertebral artery injury. Levels 0 and 1 were considered to represent accurate screw placement.

Using deviation analysis in Mimics software, the transverse and sagittal angles of the pre- and postoperative trajectories were measured and compared 7. Transverse angle: the angle between the CT-reconstructed trajectory and the sagittal midline. Sagittal angle: the angle between the left view of the CT-reconstructed trajectory and the endplate of the vertebra.

The VAS score for cervical and shoulder pain was recorded 1 d before, 3 d after and six months after surgery. In this scoring system (with a full score of 10), 0 represents no pain, and 10 represents insufferable pain. The JOA score was used (with a full score of 17) to evaluate the patient’s cervical nerve function; in this system, the higher the score, the better the nerve function.

## RESULTS

### Assessment of surgical results

The surgeries on all 17 patients were successful, with a mean procedure time of 106±25 min and a mean operative blood loss of 220±125 ml. Sixty-eight pedicle screws were inserted successfully, including 34 screws in the atlas and 34 in the axis. After surgery, there were no nerve or vertebral artery injuries or complications, including incision infections or cerebrospinal fluid leaks. During the follow-up period, there were no indications of screw loosening or breakage. *In vitro* testing was performed to compare the accuracy of screw placement with the modified 3D-printed navigation template with that of the traditional freehand, C-arm-guided and navigation-guided techniques. The results showed that the accuracy of the 3D navigation template was 0.992, which was significantly higher than that of the freehand (0.962), C-arm-guided (0.954) and navigation-guided (0.941) techniques.

### Assessment of screw placement accuracy

According to the Kawaguchi standard, 97.06% of the 68 successfully placed pedicle screws were in grade 0 (66/68), and 2.94% were in grade 1 (2/68). The success rate was 100% (Table 1). Mimics software was used to measure and compare the transverse and sagittal angles preoperatively and postoperatively (Tables 2 and 3).

The results showed that there were no significant differences in the transverse or sagittal angle preoperatively or postoperatively (mean P>0.05, Table 1).

### Postoperative nerve function assessment

The postoperative VAS score significantly decreased from 6.42±2.21 to 3.15±1.26 3 d after surgery and to 2.05±1.56 after 6 months. The postoperative JOA score significantly increased from 7.68±2.51 to 11.65±2.72 3 d after surgery and to 13.65±2.57 after 6 months. No spinal nerve injuries occurred (Table 4).

## DISCUSSION

### Clinical application and limitations of 3D-printed navigation template

Posterior atlantoaxial pedicle screw fixation has become the “gold standard” for atlantoaxial fixation. Due to the complex structure of the atlantoaxial area, traditional surgical methods pose great risks to vascular and nervous tissue. Therefore, improving the accuracy of atlantoaxial pedicle screw placement has become an urgent need for spinal surgeons. The use of various computer-aided methods can improve screw placement accuracy and safety, thereby reducing the risk of spinal cord and vascular injury 8. Although computer-aided methods have unparalleled advantages over traditional ones, there are also several disadvantages. For example, an expensive navigation system is required, the procedure complexity is increased, there is a long learning cycle for the technique, and intraoperative changes in the patient’s position are necessary. With the continuous progress being made in computer technology and the emergence of 3D printing technology, the optimum screw trajectory can be defined using reverse engineering and computer-aided technology, and when combined with a well-designed 3D-printed navigation template, can direct the pedicle screw during placement, thereby improving screw placement accuracy and reducing both the surgical risks and complication rates. Lu et al. 9 and Kawaguchi et al. 10 conducted cervical pedicle screw placement with a channel-type navigation template, and the therapeutic effect was satisfactory, without neurovascular damage. This heralded a tremendous advance in cervical treatment. However, the research 11,12 also showed that there were some limitations, as follows: ① errors may occur when producing the navigation template; ② placement deviations may be generated when the navigation template is not closely attached to the bone posterior to the vertebral lamina; ③ placement deviations may be produced when there is inadequate dissection of the muscles posterior to the vertebral lamina, spinous process and posterior arch and of soft tissues, such as ligaments; ④ 3D-printed navigation templates mainly composed of UV-curable resin were fragile after production and inclined to be unstable during application; and ⑤ disinfection and other procedures often lead to template deformation. Moreover, the researchers indicated that the surgery would be affected if the guide channels were too long due to the thick muscles in the neck and the large angles between the pedicles on both sides. In contrast, given an overly short channel, the electric drill does not run completely parallel with the channel such that cuttings would be produced at the base of the template. Therefore, placement deviations would result, along with a worn template. Furthermore, with the template being small and not conducive to support and fixation, it would be unstable and cause difficulties for surgeons in observing the template and guide tracks. Placement deviation was likely to be produced with the occurrence of subtle shaking and displacement. Jiang et al. 13 inserted 304 screws into the cervical pedicles of 39 patients with a post-type 3D-printed navigation template, and 287 screws were in grade 0 (94.4%), 15 screws were in grade 1 (4.9%), 2 screws were in grade 2 (0.7%), and none were in grade 3. The post-type template replaced the channels on both sides with location holes, guide posts and guide channels with internally shifted guide posts. This approach facilitated observation of the direction of the template and reduced the possibility of shaking and displacement of the template resulting alteration in the direction of the screw placement. Experienced spinal surgeons are indispensable, however, because the direction of the template can be easily changed following removal of the guiding channels; if this occurs, manual adjustment is needed.

### Advantages of modified 3D-printed navigation template over traditional techniques

Compared with traditional techniques, the modified 3D-printed navigation template has channels on both sides, as well as internally shifted guide posts with a length of 30 mm and diameter of 3 mm. The modified 3D-printed navigation template has many advantages over traditional techniques in atlantoaxial pedicle screw placement, the most notable of which are great improvements in accuracy and safety. The modified 3D-printed navigation template also exhibited the following advantages: ① reduced placement deviation, more accurate screw placement and intraoperative identification of template shaking and displacement via observation of the post position; ② screw placement deviation avoidance via the use of an electric drill parallel with the channel using the guide posts as a reference; ③ improved intraoperative template stability and reduced intraoperative template shaking and displacement via assistants holding the additional long posts acting as guides at opposite sides to repair the navigation template position; and ④ reduced required intraoperative space via limiting the space expansion of internally shifted guide posts on both sides, leading to less tissue traction and dissection. Therefore, screw placement deviation can be reduced if the template is fully attached to the posterior vertebral parts, as ensured by post-oriented full dissection of the posterior soft tissues.

A preoperative simulation surgery was conducted using the 3D-printed template in this study. Kirschner wire was drilled through the guide channel of the modified 3D-printed navigation template and the pedicle. Kirschner wire was inserted completely into the pedicle, and the cortex of the pedicle was not broken. The *in vitro* test demonstrated the accuracy of the modified 3D-printed navigation template. Once the feasibility of the treatment was assured, the modified 3D-printed template was disinfected and sterilized before use in patients. Pedicle screw placement assisted by the modified 3D-printed navigation template was conducted in 17 patients. The mean surgery duration for treating atlantoaxial dislocation and deformity was 106±25 min and 186±125 min, respectively; the mean operative blood loss was 220±125 ml and 312±207 ml, respectively. These results demonstrate the advantages of shorter operation times and decreased blood loss compared to traditional surgery, which is in agreement with the results of the study conducted by Hu et al. 14. All procedures were performed prior to surgery to conform with the principle of personalized treatment, including thin-slice CT scanning, medical image sampling and computerized 3D reconstruction of the atlantoaxial area, and optimal design of the pedicle screw trajectory. Sixty-eight pedicle screws were inserted successfully, including 34 screws in the atlas and 34 in the axis. According to the Kawaguchi standard, there were 97.06% screws in grade 0 (66/68) and 2.94% in grade 1 (2/68). The success rate was 100%. The preoperative transverse angles of the atlas and axis pedicle screws were left 9.0±1.6 and right 8.7±1.8 and left 29.3±6.6 and right 30.3±6.5, respectively. The postoperative transverse angles of the atlas and axis pedicle screws were left 8.8±1.3 and right 8.5±1.6 and left 29.8±6.5 and right 31.0±5.7, respectively. The preoperative sagittal angles of the atlas and axis pedicle screws were left 8.9±1.6 and right 8.7±1.4 and left 23.8±5.8 and right 24.2±6.0, respectively. The postoperative sagittal angles were left 8.9±1.5 and right 8.7±1.4 and left 23.5±5.81 and right 23.0±5.6, respectively. There were no significant differences between the preoperative or postoperative transverse or sagittal angles. This method can improve the accuracy and safety of screw placement by precisely setting the insertion site and angle 15,16. This finding agrees with the results of the studies conducted by Kawaguchi et al. 17 and Hu et al. 18. The postoperative VAS score was significantly decreased from 6.42±2.21 to 3.15±1.26 and 2.05±1.56. The postoperative JOA score increased from 7.68±2.51 to 11.65±2.72 and 13.65±2.57. These results indicate that it is safe and feasible to use a modified 3D-printed navigation template to assist in atlantoaxial pedicle screw placement.

### Shortcomings of modified 3D-printed navigation template and cautions

The modified 3D-printed navigation template also has limitations similar to those of traditional methods. ① With UV-curable resin being the main material, the 3D-printed navigation template was fragile after production, resulting in cuttings forming at the base of the template. Therefore, more attention should be focused on reducing screw placement deviation by enabling an electric drill to run parallel with the guide channels. In this research, the diameter of the guide channels, 2.1 mm, matched the drill bit diameter. Additionally, the paraffin oil used in the channels for lubrication intraoperatively reduced obstruction resistance and avoided template wear caused by the friction of the drill against the channels. ② High-temperature disinfection and other operations can lead to template deformation; therefore, ethylene oxide is recommended for both disinfection and sterilization. ③ More complex preoperative designs should be considered. ④ Deviations may occur in template production; however, we conducted preoperative experiments *in vitro* on the model to ensure that the screw trajectory did not deviate from the posterior pedicle before clinical application. Additionally, probes were used for investigating the walls of the screw placement channels, and screw placement was evaluated to define screw positions for safe surgery. ⑤ Placement deviation may be generated if the navigation template is not closely attached to the bones posterior to the vertebral lamina; therefore, it is of great importance to allow the template to closely attach to the bones by the complete removal of the corresponding spinous process, lamina, lateral mass and dorsal soft tissues. Furthermore, pedicle screw fixation is not suggested for an atlantoaxial pedicle with a diameter less than 3.5 mm.

In conclusion, this study demonstrates that it is safe and feasible to perform atlantoaxial pedicle screw placement with high accuracy and no neurovascular injuries using a modified 3D-printed navigation template. These results indicate that use of the modified 3D-printed navigation template is an acceptable new method for the localization and orientation of atlantoaxial pedicle screw placement, which is of great clinical value. However, due to the small number of samples in this study, further clinical research with larger samples and multiple centers is warranted.

## AUTHOR CONTRIBUTIONS

Each author has made an important scientific contribution to the study and has assisted with the drafting or revising of the manuscript.

## Figures and Tables

**Table 1:** Assessment of the accuracy of 17 atlantoaxial pedicle screw placements by the Kawaguchi standard.

Segments Screws		Level 0	Level I	Level II	Level III
Accuracy Rates					
C1 100	34	32	2	0	0
C2 100	34	34	0	0	0
Total 100	68	66	2	0	0

**Table 2:** Comparison of preset and actual atlas transverse and sagittal angle trajectories (*x̅*±s).

Screw Placement	Actual Trajectory		Preset Trajectory	
Angles	Left	Right	Left	Right
Transverse	8.8-1.3	8.5-1.6	9.0-1.6	8.7-1.8
Sagittal	8.6-1.5	8.7-1.4	8.9-1.6	8.7-1.4

Notes: Comparison between actual and preset trajectories, *p*>0.05.

**Table 3:** Comparison of preset and actual axis transverse and sagittal angle trajectories (*x̅*±s).

Screw Placement	Actual Trajectory		Preset Trajectory	
Angles	Left	Right	Left	Right
Transverse	29.8-6.5	31.0-5.7	29.3-6.6	30.3-6.5
Sagittal	23.5-5.1	23.0-5.6	23.8-5.8	24.2-6.0

Notes: Comparison between actual and preset trajectories, *p*>0.05.

**Table 4:** VAS and JOA scores of 17 patients the day before surgery, three days after surgery, and six months after surgery (*x̅*±s).

Time	VAS score	JOA score
1 d before surgery	6.42-2.21	7.68-2.51
3 d after surgery	3.15-1.26	11.65-2.72
Last follow-up	2.05-1.56	13.65-2.57

VAS score: preoperative and three days after surgery, preoperative and 6 months after surgery, *p*<0.05; statistically significant differences exist. Three days after surgery and six months after surgery, *p*<0.05. Statistically significant differences exist. JOA score: preoperative and three days after surgery, preoperative and six months after surgery, *p*<0.05. Statistically significant differences exist. Three days after surgery and six months after surgery, *p*<0.05. Statistically significant differences exist.
